# Multi-Scale Effects of Meteorological Conditions and Anthropogenic Emissions on PM2.5 Concentrations over Major Cities of the Yellow River Basin

**DOI:** 10.3390/ijerph192215060

**Published:** 2022-11-16

**Authors:** Jiejun Zhang, Pengfei Liu, Hongquan Song, Changhong Miao, Jie Yang, Longlong Zhang, Junwu Dong, Yi Liu, Yunlong Zhang, Bingchen Li

**Affiliations:** 1 Key Research Institute of Yellow River Civilization and Sustainable Development & Collaborative Innovation Center on Yellow River Civilization of Henan Province, Henan University, Kaifeng 475004, China; 2 College of Geography and Environmental Science, Henan University, Kaifeng 475004, China; 3 Institute of Urban Big Data, Henan University, Kaifeng 475004, China; 4 Key Laboratory of Geospatial Technology for the Middle and Lower Yellow River Regions (Henan University), Ministry of Education, Kaifeng, 475004, China; 5 College of Resource Environment and Tourism, Capital Normal University, Beijing 100048, China

**Keywords:** air pollution, PM2.5, GeoDetector model, interactive effects, the Yellow River Basin

## Abstract

The mechanism behind PM2.5 pollution is complex, and its performance at multi-scales is still unclear. Based on PM2.5 monitoring data collected from 2015 to 2021, we used the GeoDetector model to assess the multi-scale effects of meteorological conditions and anthropogenic emissions, as well as their interactions with PM2.5 concentrations in major cities in the Yellow River Basin (YRB). Our study confirms that PM2.5 concentrations in the YRB from 2015 to 2021 show an inter-annual and inter-season decreasing trend and that PM2.5 concentrations varied more significantly in winter. The inter-month variation of PM2.5 concentrations shows a sinusoidal pattern from 2015 to 2021, with the highest concentrations in January and December and the lowest from June to August. The PM2.5 concentrations for major cities in the middle and downstream regions of the YRB are higher than in the upper areas, with high spatial distribution in the east and low spatial distribution in the west. Anthropogenic emissions and meteorological conditions have similar inter-annual effects, while air pressure and temperature are the two main drivers across the whole basin. At the sub-basin scale, meteorological conditions have stronger inter-annual effects on PM2.5 concentrations, of which temperature is the dominant impact factor. Wind speed has a significant effect on PM2.5 concentrations across the four seasons in the downstream region and has the strongest effect in winter. Primary PM2.5 and ammonia are the two main emission factors. Interactions between the factors significantly enhanced the PM2.5 concentrations. The interaction between ammonia and other emissions plays a dominant role at the whole and sub-basin scales in summer, while the interaction between meteorological factors plays a dominant role at the whole-basin scale in winter. Our study not only provides cases and references for the development of PM2.5 pollution prevention and control policies in YRB but can also shed light on similar regions in China as well as in other regions of the world.

## 1. Introduction

With the acceleration of industrialization and urbanization, China’s economy has grown steadily; at the same time, the emissions of greenhouse gases and air pollutants have also continued to increase [1,2,3]. As the most urgent threat to public health and the ecosystem, PM2.5 (particulate matter of less than 2.5μm in diameter) has become a major air pollutant in China [4,5,6,7,8,9]. In order to effectively deal with the serious PM2.5 pollution problem, since 2013, the Chinese government has implemented a series of air pollution prevention and control measures that have achieved considerable results [10,11]. However, the pollution factors of PM2.5 concentrations are complex and there is variability at different spatial and temporal scales, which leads to uncertainty in the effectiveness of the implementation of air pollution prevention and control measures [12]. Therefore, conducting an analysis of PM2.5 pollution impact mechanisms, especially the performance of PM2.5 concentrations and impact factors in typical regions at different spatial and temporal scales, will have implications for the formulation of precise air pollution prevention and control policies. 

PM2.5 concentrations are affected by a variety of factors, including natural and socioeconomic factors [3,11]. Meteorological conditions (MC_S_) are important factors affecting the accumulation, dispersion, and chemical processes of pollutants. Temperature [13,14,15,16], relative humidity [17,18,19], precipitation [15,20,21], wind speed [22], sunshine duration [23], and surface air pressure [14] have significant effects on PM2.5 concentrations. In addition, anthropogenic emissions (AE_S_) from industrial, transportation, agricultural, and residential sectors also have a significant influence on PM2.5 concentrations. Anthropogenic precursors, such as sulfur dioxide, nitrogen oxide, volatile organic compounds, and ammonia, aggravate PM2.5 pollution by generating secondary aerosols through a series of photochemical reactions [12,16,24,25]. Numerous studies have also analyzed the impact of PM2.5 concentrations in terms of urbanization [26,27,28], urban form [29,30,31,32], and industrial structure [33,34].

The Chinese government has been publicly releasing air pollution site-monitoring data since 2013, which has made it possible for scholars to conduct air pollution-related studies at a national scale [3,35,36]. Meanwhile, some scholars have also conducted analyses focusing on developed regions with serious air pollution, such as Beijing-Tianjin-Hebei in northern China [12,16,37,38,39], the Yangtze River Delta in central China [40,41,42,43], the Pearl River Delta in southern China [44,45,46,47], and the Chengdu-Chongqing urban agglomeration in southwest China [21,48,49]. 

The Yellow River Basin (YRB) is an important energy, chemical, raw material, and general industrial base in northern China, as well as an ecological corridor connecting the Qinghai–Tibet Plateau, the Loess Plateau, and the North China Plain. With its accelerated urbanization, the YRB faces serious air pollution issues, mainly due to particulate matter [50]. The Fenwei Plain in the YRB became one of the three key areas for air pollution control in China in 2018, replacing the Pearl River Delta [51]. In recent years, some scholars have explored the spatial and temporal characteristics and driving mechanisms behind air pollution, focusing on key cities [26,52,53,54], major regions, and urban agglomerations [51,55,56,57] in the YRB. Wang et al. [58] studied the causes of heavy haze pollution in Xi’an, Shaanxi Province, and found that high-intensity anthropogenic emissions, relative humidity, consistent low temperature, and surface air pressure were the main drivers of heavy pollution formation. He et al. [59] studied the characteristics and source resolution of water-soluble inorganic ion species in PM2.5 in Taiyuan, Shanxi Province, China, and found that Mg^2+^ and Ca^2+^ were derived not only from soil dust but also from coal combustion and industrial emissions. Meanwhile, wind speed also affected the transport of dust and, thus, had an impact on PM2.5 and water-soluble inorganic ions. In general, scholars have conducted studies on scientific issues related to atmospheric pollution in the YRB. However, due to the complexity of the natural geographical conditions and anthropogenic emissions in the YRB, the impact mechanisms of air pollution have still not been sufficiently discussed. Most of the current studies have conducted a relative analysis based on one aspect of the natural and socioeconomic factors at a single spatial and temporal scale. However, analyses that explore the multi-scale mechanisms influencing PM2.5 concentrations in the YRB, which integrate natural and socioeconomic factors, are still lacking.

Therefore, we took the YRB as the study area and used the GeoDetector model to investigate the multi-scale effects of meteorological conditions and anthropogenic emissions and their interactions on PM2.5 concentrations. The main objectives of this research are as follows: (1) to identify the spatial and temporal distribution patterns of PM2.5 concentrations in the YRB; (2) to elucidate the effects of meteorological conditions and anthropogenic emissions on PM2.5 concentrations at different spatial and temporal scales; (3) to analyze the effect of the interaction between meteorological conditions and anthropogenic emissions on PM2.5 concentrations. Our study could provide insight into the mechanism of PM2.5 pollution in the YRB and bear out the formulation of air pollution prevention and control policies, as well as a reference for air pollution management in similar basins in China and other developing countries.

## 2. Materials and Methods

### 2.1. Study Area

**Figure 1:**
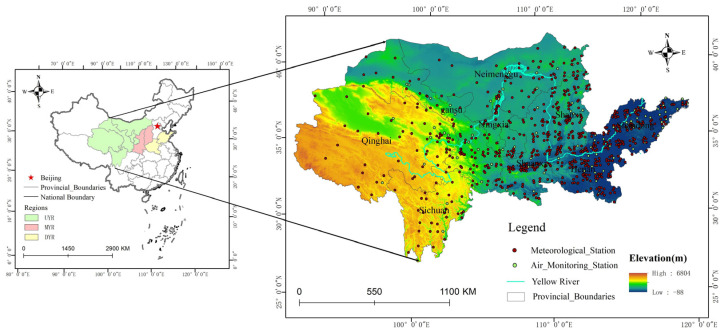
Overview of the Yellow River Basin in China. (YRB denotes the Yellow River Basin; UYR denotes the Upper Yellow River Basin; MYR denotes the Middle Yellow River Basin; DYR denotes the downstream area of the Yellow River Basin).

The YRB is located in the north of China, with a total length of 5464 km. It holds a very important position in terms of economic development and ecological security, as an important ecological barrier, and as an important economic belt in China. We have adopted the Yellow River Basin boundary identified by the Chinese National Strategy for Ecological Protection and Quality Development, involving 91 prefectural-level administrative regions. The region includes the entirety of seven provinces in Qinghai, Gansu, Ningxia, Shaanxi, Shanxi, Henan, and Shandong, as well as six cities and one union in western Inner Mongolia and two states in Sichuan, Aba, and Ganzi (Figure 1). We divided the YRB into three sub-basins, based on provincial administrative units: the Upper Yellow River Basin (UYR), including 7 cities (leagues) in Qinghai, Gansu, Ningxia, and Inner Mongolia and 2 states in Sichuan, the Middle Yellow River Basin (MYR), including Shanxi and Shaanxi, and the downstream area of the Yellow River Basin (DYR), including Henan and Shandong.

The YRB spans four geomorphic units from west to east, including the Tibetan Plateau, the Inner Mongolia Plateau, Loess Plateau, and the North China Plain, with high terrain in the west and low terrain in the east. The average elevation in the western YRB is above 4000 m, with perennial snow and glacial landform development. The central region of the YRB is between 1000 and 2000 m above sea level, with loess landforms and severe soil erosion. The eastern YRB mainly consists of the alluvial plain of the Yellow River. The Yellow River Basin has a temperate monsoon climate, with an annual precipitation of 144–843 mm, and, in most places, it is 200–600 mm. Precipitation shows a pattern with more in the southeast and less in the northwest, and it gradually decreases from the southwest to the northeast. The YRB is an important energy, chemical, raw material, and general industrial base in northern China that has been facing an air pollution problem, primarily from particulate matter, in recent years. According to the Chinese ecological environment status bulletin in 2019, of the top 20 cities with poor air quality in the country, 13 cities are located in the YRB, mainly in the central and eastern regions of the YRB and the North China Plain.

### 2.2. Data

We selected meteorological conditions and anthropogenic emissions as two types of influencing factors when conducting our analysis. The daily average meteorological data included six meteorological factors; these were accumulated precipitation (PRE, mm), surface air pressure (PRS, hPa), 2-meter relative humidity (RHU, %), sunshine duration (SSD, h), air temperature (TEM, °C) and 10-meter wind velocity (WIN, m/s). The anthropogenic emissions included five anthropogenic factors, namely, ammonia (NH_3_), nitrogen oxide (NOx), primary PM2.5 (P_PM), volatile organic compounds (VOC), and sulfur dioxide (SO_2_).

Daily average PM2.5 concentrations (2015–2021) were derived from the National Environmental Monitoring Center, which included 104 city-monitoring sites in the YRB. Daily average meteorological monitoring data (2015–2017) were obtained from the China Meteorological Data Network (http://data.cma.cn/ (accessed on 11 April 2022)) (Figures S1–S6 in the Supplementary Materials), which included 686 meteorological monitoring sites in the YRB. Anthropogenic emissions were derived from the Tsinghua University Emission Inventory (2015–2017) (MEIC, http://meicmodel.org/ (accessed on 11 April 2022)) (Figures S7–S11 in the Supplementary Materials). The raster resolution of the inventory is 0.25° × 0.25°, and the temporal resolution includes the year and month [6,60].

### 2.3. GeoDetector Model

The GeoDetector model is a tool to detect and utilize spatial heterogeneity. This method has no linear assumption and has a clear physical meaning, which means that the multi-collinearity of input factors can be eliminated or ignored [61]. The GeoDetector model includes four detectors, comprising factor detection, interaction detection, risk area detection, and ecological detection. In our study, factor detection and interaction detection were used to quantify the effects of MC_S_ and AEs and their interactions on PM2.5 concentrations in the YRB.

Factor detectors can express the extent to which the independent variables explain the spatial differentiation of the dependent variables through q. In our study, the meteorological emission factors (PRE, PRS, RHU, SSD, TEM, and WIN) and anthropogenic emission precursors (NH_3_, NO_X_, P_PM, SO_2_, and VOC) were selected as the independent variables (X), with PM2.5 concentrations as the dependent variables (Y). The numerical variables were stratified and converted to categorical variables before implementing the variables. After comparing various classification methods, our study used the quantile method to classify each variable into 8 classes. These are expressed as:

(1) $$q=1-\frac{\sum_{h=1}^{L}N_{h}\sigma_{h}{}^{2}}{N\sigma^{2}}=1-\frac{SSW}{SST}$$

(2) $$SSW=\overset{L}{\underset{h=1}{\sum }}N_{h}\sigma_{h}{}^{2}$$

(3) $$SST=N\sigma^{2}$$

The q value, which is between 0 and 1, represents the effect of the driving factor on spatial heterogeneity in PM2.5 concentrations. If the spatial distribution of PM2.5 concentrations is completely determined by a particular factor X, the q-value is 1; however, if there is no spatial correlation between PM2.5 concentrations and factor X, then the q-value is 0. In the formula (1), *h* = 1… and L represents the number of sub-regions of the influential factors; N and $N_{h}$ are the numbers of the whole region and sub-regions in classification *h*; σ and $\sigma_{h}$ represent the total variance and the variance of samples from sub-regions, respectively.

The interaction detector is used to identify the explanatory power of the interaction between any two influencing factors (X1 ∩ X2) on the dependent variable (Y). We calculated the *q*-value of the interaction between any two factors (X1 ∩ X2) on Y and compared it with the *q*-values of X1 and X2. The interaction between any two factors falls into five categories, comprising nonlinearly weakened, one-factor nonlinearly weakened, two-factor-enhanced, independent, and nonlinearly enhanced categories. For details, please refer to the literature [62].

## 3. Results

### 3.1. Temporal Variation of PM2.5 Concentrations

**Figure 2:**
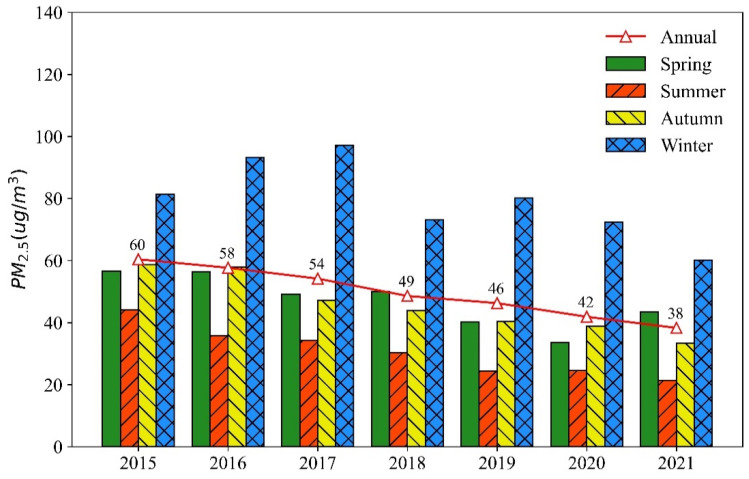
Inter-annual and inter-season variations of PM2.5 concentrations in the Yellow River Basin from 2015 to 2021.

Figure 2 shows the annual and seasonal variations in PM2.5 concentrations in the YRB from 2015 to 2021. From 2015 to 2021, the inter-annual variation of PM2.5 concentrations in the YRB demonstrates a decreasing trend, from 60 µg/m^3^ in 2015 to 38 µg/m^3^ in 2021. In terms of inter-season scale, the highest average PM2.5 concentrations occurred in winter, followed by spring and autumn, while the lowest PM2.5 concentrations occurred in summer. The highest PM2.5 concentrations occurred in winter 2017, at 97 µg/m^3^, and the lowest, at 21 µg/m^3^, in summer 2021. In general, the seasonal variation of PM2.5 concentrations in the YRB presents a fluctuating downward trend from 2015 to 2021. In addition, compared with other seasons, the trend in PM2.5 concentrations is more significant in winter.

**Figure 3:**
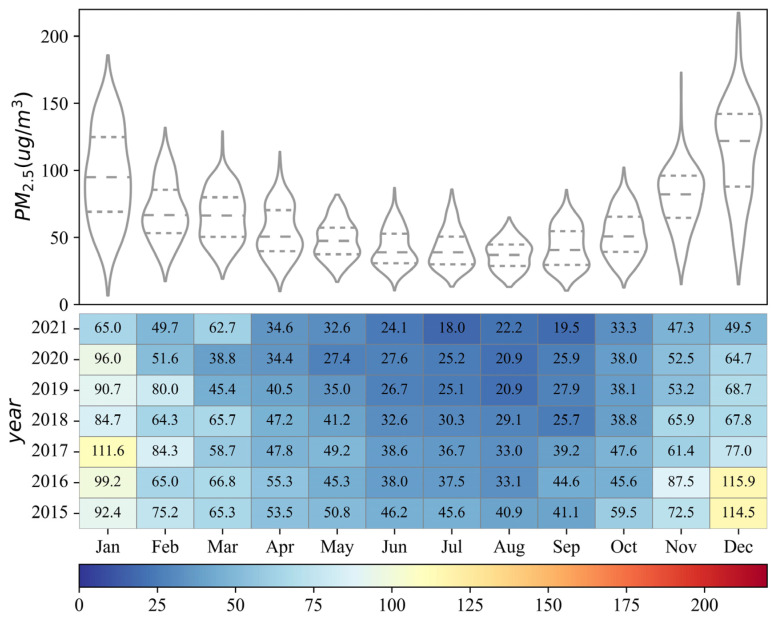
Inter-month variations of PM2.5 concentrations in the Yellow River Basin from 2015 to 2021.

Figure 3 shows the inter-month variation of PM2.5 concentrations in YRB from 2015 to 2021. The PM2.5 concentrations in the YRB display significant inter-month variation, with the lowest PM2.5 concentration value being 18 µg/m^3^ in July 2021 and the highest PM2.5 concentration value being 115.9 µg/m^3^ in December 2016. The inter-month variation of PM2.5 concentrations shows a sinusoidal pattern from 2015 to 2021, with a decreasing trend from January to May, a stable period from June to August, and an increasing trend from September to December. The fluctuation in monthly average PM2.5 concentrations from 2015 to 2021 demonstrates that the fluctuation range is the largest in January and December and the smallest in August. The kernel density curve shows that the curve for August is the steepest, with its peak corresponding to lower PM2.5 concentrations than other months, indicating that PM2.5 concentrations in major cities in the YRB are generally lower in August. However, the kernel density curves in December and January are flatter and have the most peaks, indicating that PM2.5 concentrations in major cities in the YRB are mostly high during this period, with a high degree of internal polarization.

### 3.2. Spatial Variations of PM2.5 Concentrations

**Figure 4:**
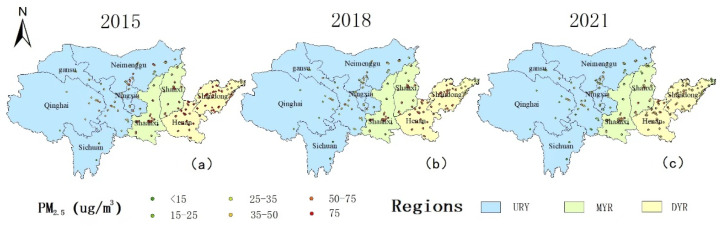
Spatial distribution of PM2.5 annual average concentrations in the Yellow River Basin from 2015 to 2021 (**a**–**c**). (UYR denotes the Upper Yellow River Basin, MYR denotes the Middle Yellow River Basin, and DYR denotes the downstream area of the Yellow River Basin).

The spatial distribution of the annual average PM2.5 concentrations in the YRB from 2015 to 2021 is shown in Figure 4. There are obvious differences in the spatial distribution. From 2015 to 2021, the annual average PM2.5 concentrations in the YRB display a significant downward trend. In 2015, the heavily polluted areas were mainly concentrated in the MYR and DYR areas, including the central and western parts of Shandong, most of Henan, the central part of Shaanxi, and Shanxi. The annual average PM2.5 concentrations in these areas exceeded 50 µg/m^3^, far exceeding the China Ambient Air Quality Standard (CAAQS) Class II standard (35 µg/m^3^). Low-pollution areas were mainly concentrated in the UYR, as well as in southwestern Shanxi and the western Shandong Peninsula. Compared to 2015, PM2.5 concentrations levels decreased significantly in 2018, especially in the MYR and DYR areas. In 2018, high-pollution areas were concentrated in Henan, western Shandong, and central Shaanxi. In addition, relatively low pollution areas were mainly concentrated in western Shandong and southwest Shanxi. In 2021, PM2.5 concentrations were further reduced; the relatively high-pollution areas at this point were mainly concentrated in Shanxi, Shaanxi, Henan, and Shandong.

### 3.3. Multi-Scale Effects of Influencing Factors on PM2.5 Concentrations

**Figure 5:**
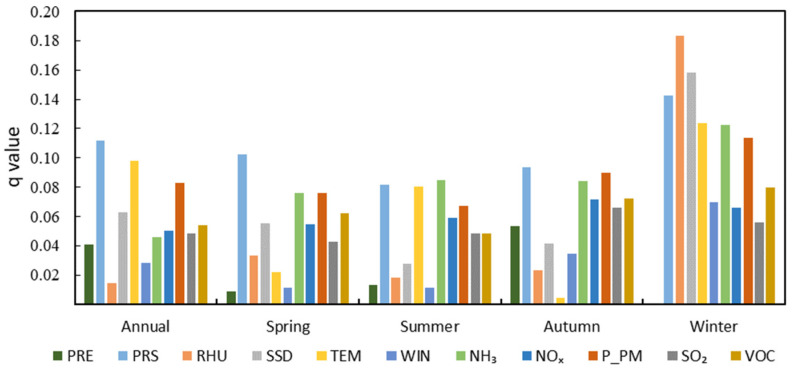
The inter-annual and inter-season *q*-values of the driving factors on PM2.5 concentrations in the Yellow River Basin. (PRE denotes the accumulated precipitation; PRS denotes the surface air pressure; RHU denotes the 2-meter relative humidity; SSD denotes sunshine duration; TEM denotes air temperature; WIN denotes the 10-meter wind velocity; NH_3_ denotes ammonia; NOx denotes nitrogen oxides; P_PM denotes primary PM2.5; SO_2_ denotes sulfur dioxide; VOC denotes volatile organic compounds).

At the whole-basin scale, the influence of driving factors on PM2.5 concentrations demonstrates large inter-annual and inter-season variations (Figure 5 and Table S1 in the Supplementary Materials). MC_S_ and AE_S_ have similar inter-annual effects on PM2.5 concentrations in YRB. PRS (*q* = 0.112), TEM (*q* = 0.098), and P_PM (*q* = 0.083) are thought to be the major influencing factors on PM2.5 concentrations. PRS plays a dominant role among MC_S_, and P_PM is the leading impactor among the AE_S_.

In spring, the dominant factor affecting PM2.5 concentrations in the YRB is PRS (*q* = 0.102), followed by NH_3_ (*q* = 0.076) and P_PM (*q* = 0.076). In summer, NH_3_ (*q* = 0.085), PRS (*q* = 0.082), and TEM (*q* = 0.080) are the three dominant factors influencing PM2.5 concentrations. In autumn, the dominant emission factors affecting PM2.5 concentrations are PRS (*q* = 0.094), P_PM (*q* = 0.090), and NH_3_ (*q* = 0.084). However, the dominant factors affecting PM2.5 concentrations in winter are RHU (*q* = 0.184), SSD (*q* = 0.158), and PRS (*q* = 0.143), respectively. In general, AE factors and MC_S_ have a similar influence on PM2.5 concentrations in spring, summer, and autumn, while MC_S_ have a greater influence on PM2.5 concentrations than AE_S_ in winter.

**Figure 6:**
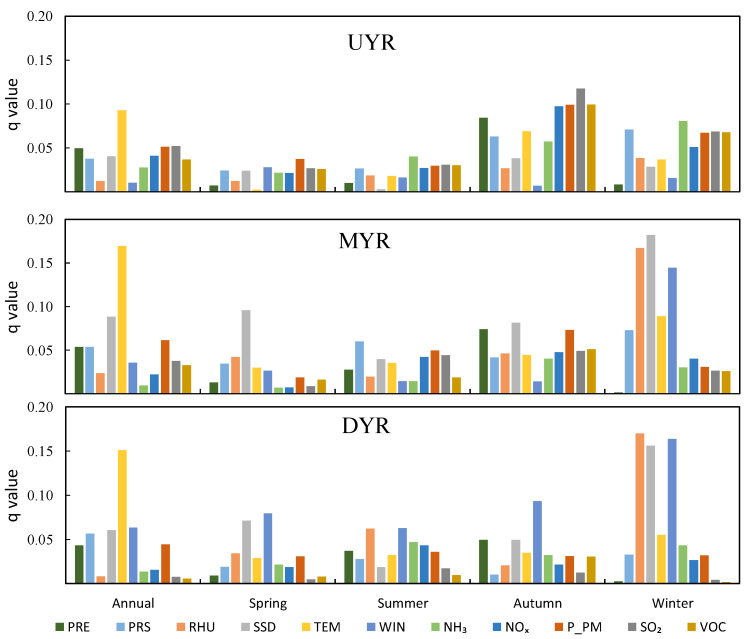
The inter-annual and inter-season *q*-values of the driving factors on PM2.5 concentrations in the sub-basin of the Yellow River Basin. (UYR denotes the Upper Yellow River Basin; MYR, denotes the Middle Yellow River Basin; DYR, denotes the downstream area of the Yellow River Basin; PRE denotes accumulated precipitation; PRS denotes surface air pressure; RHU denotes 2-m relative humidity; SSD denotes sunshine duration; TEM denotes air temperature; WIN denotes 10-m wind velocity; NH_3_ denotes ammonia; NOx denotes nitrogen oxides; P_PM denotes primary PM2.5; SO_2_ denotes sulfur dioxide; VOC denotes volatile organic compounds).

Figure 6 and Table S2 in the Supplementary Materials show that MC_S_ have a stronger inter-annual effect on PM2.5 concentrations than AE_S_ at the sub-basin scale, and TEM is the dominant influencing factor on PM2.5 concentrations. In the UYR, TEM (*q* = 0.093) is the dominant factor for PM2.5 concentrations, followed by SO_2_ (*q* = 0.052) and P_PM (*q* = 0.051). In the MYR, TEM (*q* = 0.17), SSD (*q* = 0.09), and P_PM (*q* = 0.06) are the three dominant factors affecting PM2.5 concentrations. However, the dominant factor affecting PM2.5 concentrations is TEM (*q* = 0.15), followed by WIN (*q* = 0.06) and SSD (*q* = 0.06) in the DYR. In general, TEM has similar inter-annual effects on the three sub-basins of the YRB, and the effect on PM2.5 concentrations is the largest of all the influencing factors. However, P_PM has a dominant inter-annual effect on PM2.5 concentrations among the MC_S_ in the three sub-basins.

There are significant inter-season variations in the factors influencing PM2.5 concentrations at the sub-basin scale (Figure 6 and Tables S3–S6 in the Supplementary Materials). In the UYR, MC_S_ and AE_S_ have a stronger influence on PM2.5 in autumn and winter, compared to spring and summer. AE_S_ have the greatest influence on PM2.5 concentrations in autumn, ranked first by SO_2_ (*q* = 0.12), followed by VOC (*q* = 0.10), P_PM (*q* = 0.10), and NOx (*q* = 0.10). In the MYR, MC_S_ and AE_S_ have an equal influence on PM2.5 concentrations in summer and autumn. However, MC_S_ have a stronger effect compared to AE_S_ on PM2.5 concentrations in spring and winter, and MC_S_ have the strongest effect on PM2.5 in winter for all four seasons. In the DYR, MC_S_ and AE_S_ have similar effects on PM2.5 concentrations in summer. However, MC_S_ are the dominant factors regarding PM2.5 concentrations in spring, autumn and winter, and MC_S_ are the strongest in winter. 

### 3.4. Multi-Scale Effects of Interactions on PM2.5 Concentrations

**Figure 7:**
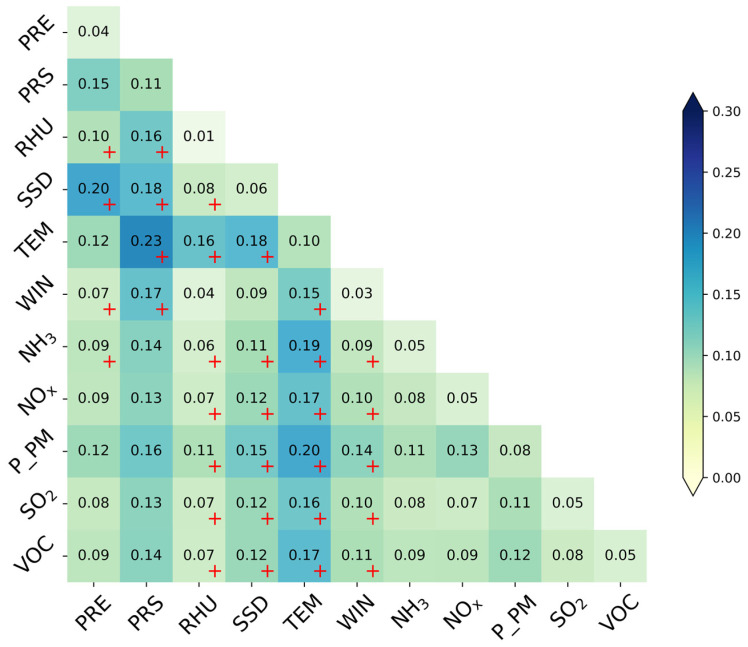
The inter-annual interaction in the factors influences the spatial pattern of PM2.5 concentrations in the Yellow River Basin. Note: “+” means that the type of interaction belongs to nonlinear enhancement; otherwise, it belongs to bivariate enhancement. (PRE denotes accumulated precipitation; PRS denotes surface air pressure; RHU denotes 2-m relative humidity; SSD denotes sunshine duration; TEM denotes air temperature; WIN denotes 10-m wind velocity; NH_3_ denotes ammonia; NOx denotes nitrogen oxides; P_PM denotes primary PM2.5; SO_2_ denotes sulfur dioxide; VOC denotes volatile organic compounds).

We explored the effect of the interaction between 55 pairs of influencing factors on PM2.5 concentrations, based on an interaction detector. The interaction between any two factors was analyzed by comparing the contribution of individual factors to PM2.5 concentrations, with the integrated contribution being between the influencing factors. Figure 7 shows the interactive *q*-values and interaction types between the influencing factors at the whole-basin scale. The interactions of 23 pairs of influencing factors are two-factor-enhanced, while the remaining 32 pairs belong to non-linear enhancement. Among them, the interaction between PRS and TEM at the inter-annual scale (*q* = 0.23) plays a dominant role in PM2.5 concentrations, followed by PRC ∩ SSD (*q* = 0.20) and TEM ∩ P_PM (*q* = 0.20). There is also a large seasonal difference in the interaction between potential influencing factors (Figure S12 in the Supplementary Materials). On a seasonal scale, the interaction between AE_S_ plays a dominant role in summer. In contrast, the interaction between MC_S_ plays a dominant role in PM2.5 concentrations in spring, autumn, and winter, with the strongest interaction between RHU and PRS in winter. There is also a significant influence between MC_S_ and AE_S_, with the strongest interaction shown between RHU and P_PM and NH_3_ in winter (*q* = 0.27).

Figures S13–S18 in the Supplementary Materials show the significant regional variations in the interactions at the sub-basin scale. In the UYR, the inter-annual interactions between MC_S_ have a similar effect as those between MC_S_ and AE_S_. In the MYR, the inter-annual interactions between MC_S_ play a dominant role in PM2.5 concentrations, with the strongest interactions occurring between TEM and SSD. Similar to MYR, the inter-annual interaction between MC_S_ plays a dominant role in terms of PM2.5 concentrations in the DYR region, with the strongest interactions between TEM and WIN. In addition, Figures S13–S18 also show the *q*-values of the inter-season interaction between the impact factors in the sub-basin, indicating that there is also significant inter-season variation in the interaction between the impact factors in the sub-basin.

## 4. Discussion

The inter-annual PM2.5 concentrations in the YRB displayed a steady downward trend from 2015 to 2021, which is consistent with a series of atmospheric pollution control policies that China has implemented in recent years. For example, the Chinese government promulgated the Air Pollution Prevention and Control Action Plan in 2013 and the Three-Year Action Plan to Win the Blue Sky Defense War in 2018. The implementation of these air-quality policies has had a significant effect on reducing PM2.5 concentrations [10,11,63,64]. The inter-season variation of PM2.5 concentrations in the YRB shows a fluctuating downward trend, and the trend of PM2.5 concentrations in winter is more obvious. At the inter-month scale, PM2.5 concentrations demonstrate a U-shaped distribution from January to December, with January and December at the peak and June to August at the bottom of the curve, which is consistent with previous studies [56,65]. In terms of spatial distribution, PM2.5 concentrations in the YRB display a spatial distribution pattern that is high in the east and low in the west. The high PM2.5 concentrations are mainly concentrated in Henan, Shandong, Shanxi, and Shaanxi, and are located in the MYR and DYR. This may be due to the dense population in the region; the related industries are mainly for energy and basic raw materials, and the energy consumption structure is mainly with coal [30,66,67].

We quantified the multi-scale effects of MC_S_ and AE_S_ and their interactions on PM2.5 concentrations in the YRB, based on a GeoDetector model. We found that there are significant inter-annual and inter-season variations in the effects of MC_S_ and AE_S_ on PM2.5 concentrations at the whole-basin scale. The inter-annual effects of MC_S_ and AE_S_ on PM2.5 concentrations are similar, and PRS and TEM are the two main factors affecting PM2.5 concentrations. TEM and PRS influence particulate matter transport and accumulation by affecting convection [21]. Air pressure is positively correlated with PM2.5 concentrations [14]. Air pressure affects the dispersion and accumulation of PM2.5 by creating low-level wind convergence. High-pressure systems can lead to environmental stagnation, which affects PM2.5 transport and is detrimental to PM2.5 dispersion [68]. Comparing relevant studies in other regions of China, previous scholars have found that air pressure also has a dominant effect on PM2.5 concentrations in Tibet and China [69,70].

A strong relationship between temperature and PM2.5 concentrations has been demonstrated in the literature [3,14,15,71]. Strong thermal activity, such as turbulence, at high temperatures will accelerate the dispersion of PM2.5 [72]. In contrast, a low temperature reduces atmospheric convection and enhances PM2.5 agglomeration. In general, temperatures decrease as altitude increases, and temperature inversion occurs when temperatures increase with altitude [73]. Temperature inversion weakens PM2.5 scattering and dispersion, resulting in higher local PM2.5 concentrations [3,74,75]. Comparing relevant studies in other regions of China, some scholars found that temperature also has a dominant effect on PM2.5 concentrations in regions such as the Yangtze River delta [76], Pearl River delta [77], and northeast China [78]. In winter, however, MC_S_ play the dominant role and RHU is the most important factor, which is consistent with the results from previous studies. One possible reason is that aqueous-phase aerosol chemistry drives secondary pollution [79,80,81,82]. The finding of RHU as the dominant influencing factor of PM2.5 concentrations has also been verified in relevant studies on the Beijing-Tianjin-Hebei region [83], the Yangtze River delta [40], and the Pearl River delta [84] in China.

We found that there are spatial and seasonal variations in the effects of anthropogenic and meteorological factors on PM2.5 concentrations in the sub-basins. MC_S_ have a stronger inter-annual influence on PM2.5 concentrations than AE_S_ in the sub-basins. TEM is the dominant influencing factor in each sub-basin, which is consistent with previous studies [39,58,59]. On the inter-season scale, MC_S_ play a dominant role in winter in the MYR and UYR, which is different from previous studies. In northern China the influence of AE_S_ on PM2.5 concentrations in winter has been found to be greater than MC_S_, which is due to high coal consumption and high industrial and vehicular emissions during the heating period, resulting in severe regional PM2.5 pollution. The Fenwei Plain is located in the MYR, and PM2.5 pollution is severe in this region. PM2.5 concentrations that are attributable to vehicle exhaust, biomass combustion, substances from coal combustion, and dust have been reported in previous studies [51,85]. WIN has a significant effect on PM2.5 concentrations in the four seasons of the DYR, with the strongest effects in winter, which finding is also consistent with previous studies [53,58,86]. Some major cities in the DYR are located in the Beijing-Tianjin-Hebei atmospheric pollution transport channel, in which wind speed affects PM2.5 transmission across the region. As a result, the formation of heavy PM2.5 pollution is driven by lower wind speeds in winter in the region. Related studies have confirmed that at low wind speeds, increasing wind speed may lead to less turbulence intensity, weak horizontal atmospheric motion, and a dominant subsidence motion in the upper air, which creates unfavorable dispersion conditions for PM2.5 [87,88,89,90].

Compared with other anthropogenic emissions, P_PM has significant effects on PM2.5 concentrations at whole-basin and sub-basin scales. In recent years, anthropogenic emissions in the YRB have shown a decreasing trend over the years, indicating that reducing anthropogenic emissions can effectively reduce P_PM emissions, while pollution control measures are effective in reducing PM2.5 pollution. We find that NH_3_ is the dominant factor regarding PM2.5 in the YRB in summer. NH_3_, as an alkaline gas, has an important influence on the formation of PM2.5, as has been demonstrated in China and elsewhere in the world [91,92,93]. Agricultural activities, as well as relatively high temperatures, result in peak agricultural ammonia emissions in summer. Henan and Shandong in the DYR are both important agricultural provinces with much higher agricultural NH_3_ emissions than other regions [10,94,95]. Among all anthropogenic ammonia emission sources in Henan Province, livestock and nitrogen fertilizer are the main sources of total NH_3_ emissions, with an average share of about 46.5% and 42.9% [96]. Previous studies have demonstrated that the mitigation of agricultural ammonia emissions can help reduce PM2.5 pollution [24], and ammonia emission reduction is more cost-effective than NOx emission reduction [52]. Compared with MC_S_, AEs are more controllable. Therefore, we should focus on the corresponding policies for ammonia emission reduction in the future, which is important for the effective mitigation of PM2.5 pollution.

The interaction detector showed that the types of interactions between the influencing factors mainly included non-linear enhancement and bivariate enhancement, with non-linear enhancement dominating, indicating that interactions between factors had a significant enhancing effect on PM2.5 concentrations. In the whole basin and sub-basin, the interaction between NH_3_ and anthropogenic emissions have a dominant effect on PM2.5 concentrations in summer, which may be due to the accelerated chemical reactions between NH_3_ and other anthropogenic emissions under higher temperature conditions, which have an impact on PM2.5 concentrations [97,98]. At the whole-basin scale, the interaction between MC_S_ plays a dominant role in winter (RHU ∩ PRS), which may be due to secondary pollution driven by aqueous-phase aerosol chemistry in the YRB in winter [79,80]. Meanwhile, low pressure is often accompanied by low wind speed and higher domestic heating emissions in winter in the YRB, which together accelerate the rapid accumulation of PM2.5 [73,87,99,100].

There are some limitations to our study at present since it is limited by data accessibility; we used PM2.5 site monitoring data from 2015 to 2021 and meteorological conditions and emission inventory data from 2015 to 2017, which could conduce some uncertainty in the results. In the future, we will update meteorological conditions and emission inventory data to be consistent with PM2.5 concentrations. In the future, we will also focus on evaluating the effectiveness of air pollution policies and the impact of the implementation of air pollution policies on PM2.5 concentrations in the YRB in recent years. Relevant studies have demonstrated that PM2.5 reduction may lead to an ozone increase, which will bring uncertainty regarding the effectiveness of policy implementation. Therefore, we will combine PM2.5 with ozone measurements to conduct research on their synergistic management, in order to provide insight into the future development of precise air pollution prevention and control policies.

## 5. Conclusions

We analyzed the multi-scale variations of PM2.5 concentrations in the YRB and elucidated the multi-scale effects of meteorological conditions and anthropogenic emissions, as well as their interactions, on PM2.5 concentrations in the YRB. The results contribute to a better understanding of the driving mechanisms of PM2.5 concentrations in the YRB and provide insight into the future development of regional air pollution policies. We verified that the PM2.5 concentrations in the YRB displayed significant spatial and temporal variations from 2015 to 2021. The inter-annual variation of PM2.5 concentrations in the YRB from 2015 to 2021 displays a steadily decreasing trend. The PM2.5 concentrations in terms of inter-season scale show a fluctuating decreasing trend, and the variation is more obvious in winter. The PM2.5 inter-month concentrations display a sinusoidal pattern from 2015 to 2021, with January and December at the peak and June to August at the bottom. The PM2.5 concentrations in the YRB demonstrate a spatial distribution pattern that is high in the east and low in the west, while the high PM2.5 concentrations are mainly concentrated in the MYR and DYR.

MCs and AEs have similar inter-annual effects on PM2.5 concentrations across the whole basin, with PRS and TEM being the two most dominant factors. In contrast, MCs play a dominant role in winter, and RHU is the most important factor. At the sub-basin scale, MCs have stronger inter-annual effects on PM2.5 concentrations than AEs, while TEM is the dominant impact factor. Wind speed has a significant effect on PM2.5 concentrations across all four seasons in the DYR and has the strongest effect in winter. P_PM and NH_3_ are the two main anthropogenic emission factors affecting PM2.5 concentrations. The interaction between NH_3_ and other anthropogenic emissions plays a dominant role in PM2.5 concentrations at the whole-basin and sub-basin scales in summer, while the interaction between meteorological factors (RHU ∩ PRS) plays a dominant role at the whole-basin scale in winter.

This study can provide a policy reference for the mitigation of PM2.5 pollution. The influence of atmospheric processes on PM2.5 concentrations is complex. We found that MCs such as PRS, TEM, WIN, and RHU have important effects on PM2.5 concentrations in the Yellow River Basin. It is important to formulate a reasonable spatial planning policy for green spaces, establish and improve ecological landscapes and conservation corridors, utilize artificial precipitation enhancement, and build urban ventilation corridors to reduce regional PM2.5 concentrations [101].

Among the AEs, the influence of P_PM and NH_3_ on PM2.5 concentrations in the Yellow River Basin is more obvious. Primary PM2.5 and secondary PM2.5 have different pathways of formation; controlling primary PM2.5 emissions is effective in mitigating PM2.5 pollution. Therefore, in the future, it will be necessary to strengthen the regulation of emission reduction measures, strengthen the management of vehicle emission control policies, promote the optimization and adjustment of high energy-consuming and high-polluting industries, adjust the energy consumption structure, and optimize industrial processes in the Yellow River Basin to reduce the emissions of primary particulate matter. In the case of ammonia, it is necessary to target agricultural ammonia sources to reduce emissions, optimize farm management patterns, improve fertilizer quality, improve fertilizer application techniques and increase fertilizer utilization efficiency. In addition, more attention should be paid to non-agricultural sources of ammonia emissions, and the model of combining cultivation and farming is the key to ammonia reduction in farming.

## Supplementary Materials

The following supporting information can be downloaded at: https://www.mdpi.com/article/10.3390/ijerph192215060/s1.

## Abbreviations

AEs: anthropogenic emissions; CO, carbon monoxide; MCs, meteorological conditions; PM2.5, fine particulate matter; NH_3_ ammonia; NOx, nitrogen oxides; P_PM, primary PM2.5; PRE, accumulated precipitation; PRS, surface air pressure; RHU, 2-meter relative humidity; SO_2_, sulfur dioxide; SSD, sunshine duration; TEM, air temperature; VOC, volatile organic compounds; WIN, 10-m wind velocity; YRB, the Yellow River Basin; UYR, the Upper Yellow River Basin; MYR, the Middle Yellow River Basin; DYR, the downstream area of the Yellow River Basin.
