# Supplementary material for: Multi-Scale Effects of Meteorological Conditions and Anthropogenic Emissions on PM2.5 Concentrations over Major Cities of the Yellow River Basin
Source: Int J Environ Res Public Health. 2022 Nov 16;19(22):15060. doi: 10.3390/ijerph192215060 (PMC9690158; doi:10.3390/ijerph192215060)
Supplement: Supplementary file 1 [file ijerph-19-15060-s001.zip › ijerph-1947032-supplementary.pdf]

## Supplementary Material

# Multi-Scale Effects of Meteorological Conditions and Anthropogenic Emissions on PM2.5 Concentrations over Major Cities of the Yellow River Basin

Jiejun Zhang [1,†], Pengfei Liu [1,2,3,*,†], Hongquan Song [2,3,4,*], Changhong Miao [1], Jie Yang [1], Longlong Zhang [2], Junwu Dong [5], Yi Liu [1], Yunlong Zhang [2], Bingchen Li [2]

1 Key Research Institute of Yellow River Civilization and Sustainable Development & Collaborative Innovation Center on Yellow River Civilization of Henan Province, Henan University, Kaifeng 475004, China

2 College of Geography and Environmental Science, Henan University, Kaifeng 475004, China

3 Institute of Urban Big Data, Henan University, Kaifeng 475004, China

4 Key Laboratory of Geospatial Technology for the Middle and Lower Yellow River Regions (Henan University), Ministry of Education, Kaifeng 475004, China

5 College of Resource Environment and Tourism, Capital Normal University, Beijing 100048, China

* Correspondence: lpf@henu.edu.cn (P.L.); hqsong@henu.edu.cn (H.S.)

† These authors contributed equally to this work and should be considered co-first authors.

## List of Figures

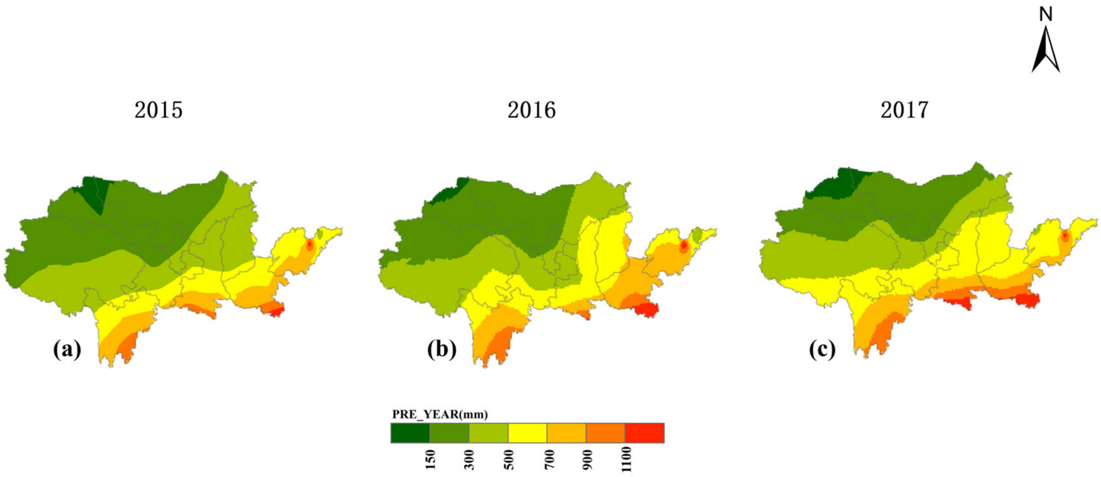


**Figure S1**. Spatial distributions of annual mean precipitation in the Yellow River Basin during 2015-2017.

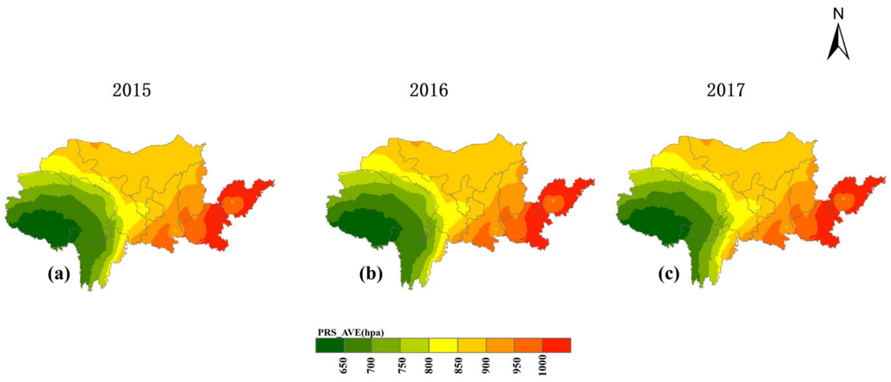


**Figure S2**. Spatial distributions of annual mean surface air pressure in the Yellow River Basin during 2015-2017.

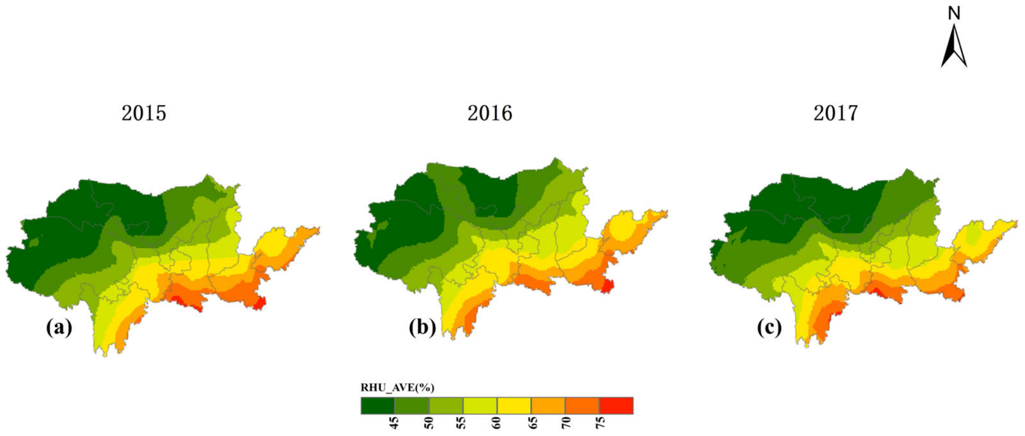


**Figure S3**. Spatial distributions of annual mean 2-m relative humidity in the Yellow River Basin during 2015-2017.

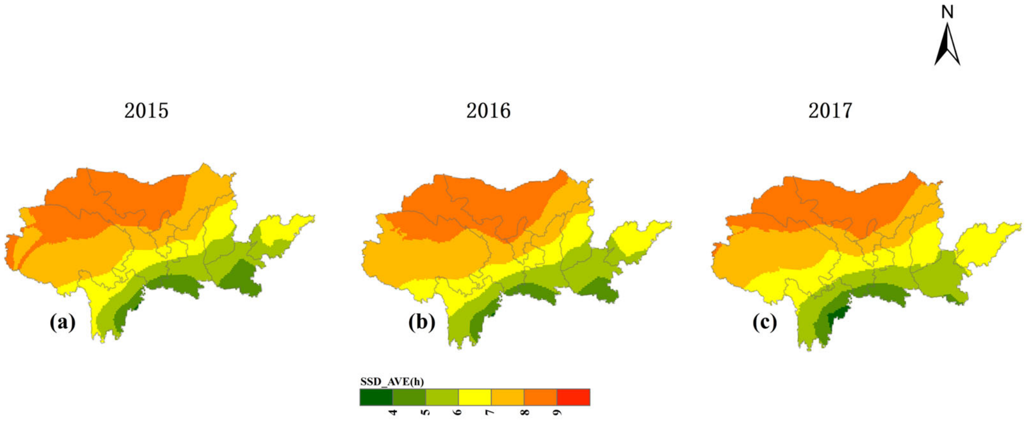


**Figure S4**. Spatial distributions of annual mean sunshine duration in the Yellow River Basin during 2015-2017.

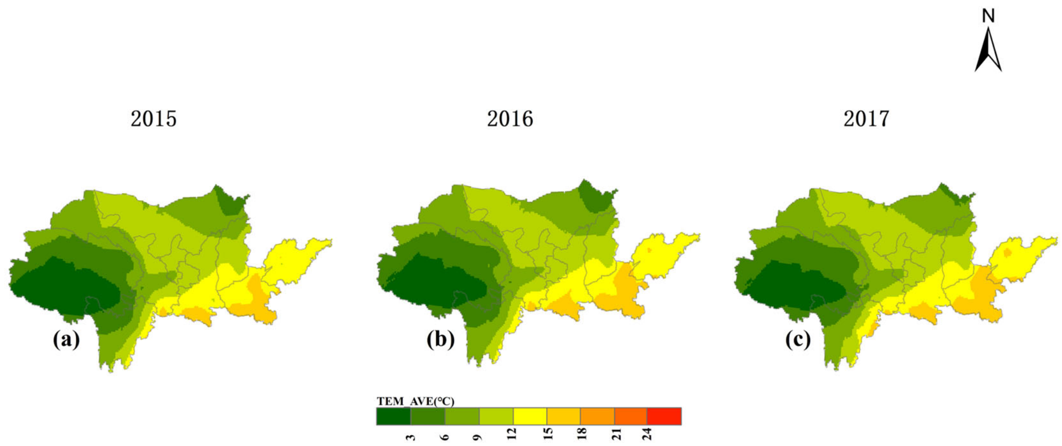


**Figure S5**. Spatial distributions of annual mean air temperature in the Yellow River Basin during 2015-2017.

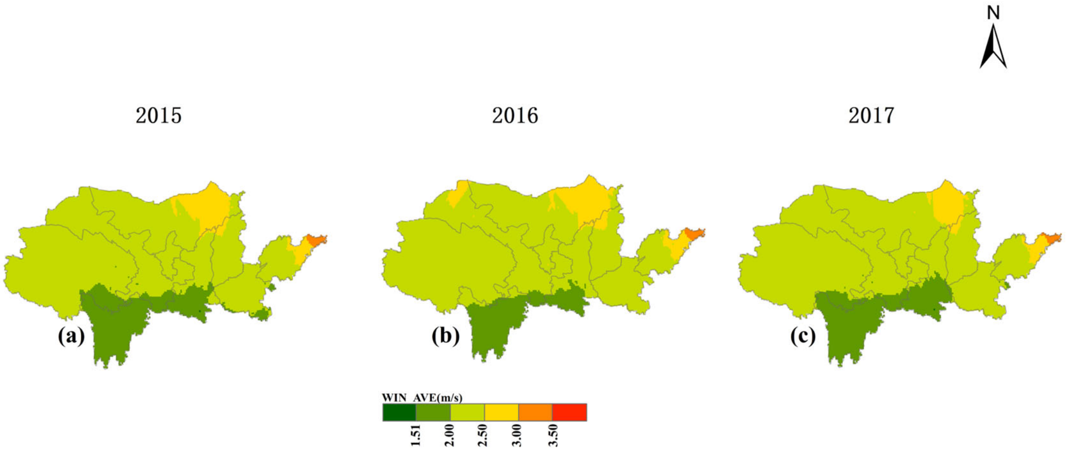


**Figure S6**. Spatial distributions of annual mean 10-m wind velocity in the Yellow River Basin during 2015-2017.

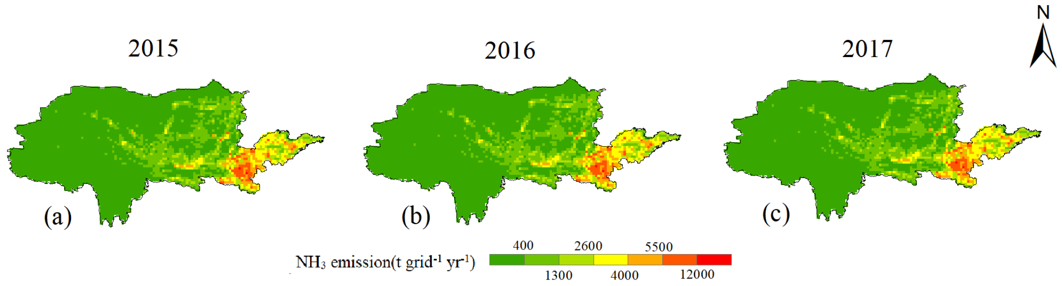


**Figure S7**. Spatial distributions of annual $NH_3$ emissions in the Yellow River Basin during 2015-2017 with resolution of 0.25° × 0.25°. ($NH_3$ denotes ammonia)

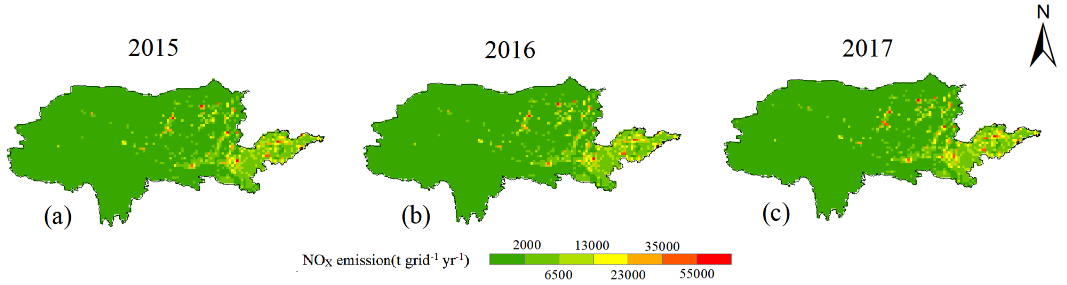


**Figure S8**. Spatial distributions of annual $NO_X$ emissions in the Yellow River Basin during 2015-2017 with resolution of 0.25° × 0.25°. (NOx denotes nitrogen oxides)

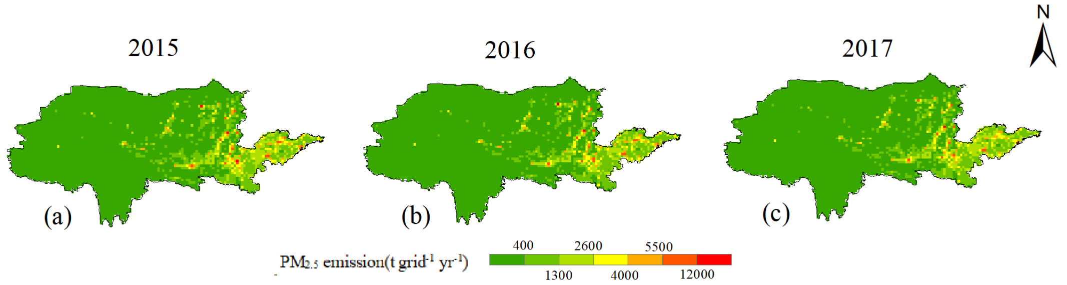


**Figure S9**. Spatial distributions of annual P_PM emissions in the Yellow River Basin during 2015-2017 with resolution of 0.25° × 0.25°. (P_PM denotes primary $PM_{2.5}$)

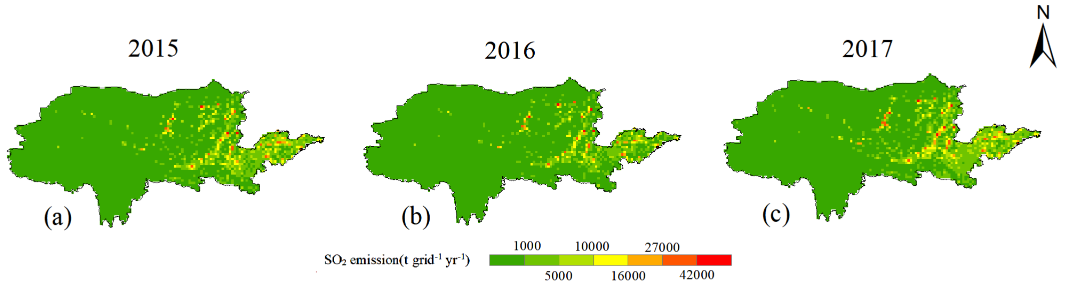


**Figure S10**. Spatial distributions of annual $SO_2$ emissions in the Yellow River Basin during 2015-2017 with resolution of 0.25° × 0.25°. ($SO_2$ denotes sulfur dioxide)

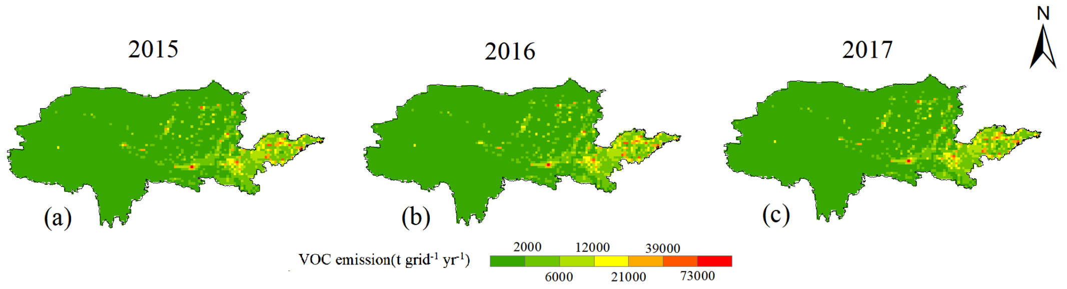


**Figure S11**. Spatial distributions of annual $VOC_S$ emissions in the Yellow River Basin during 2015-2017 with resolution of 0.25° × 0.25°. ( VOC denotes volatile organic compounds)

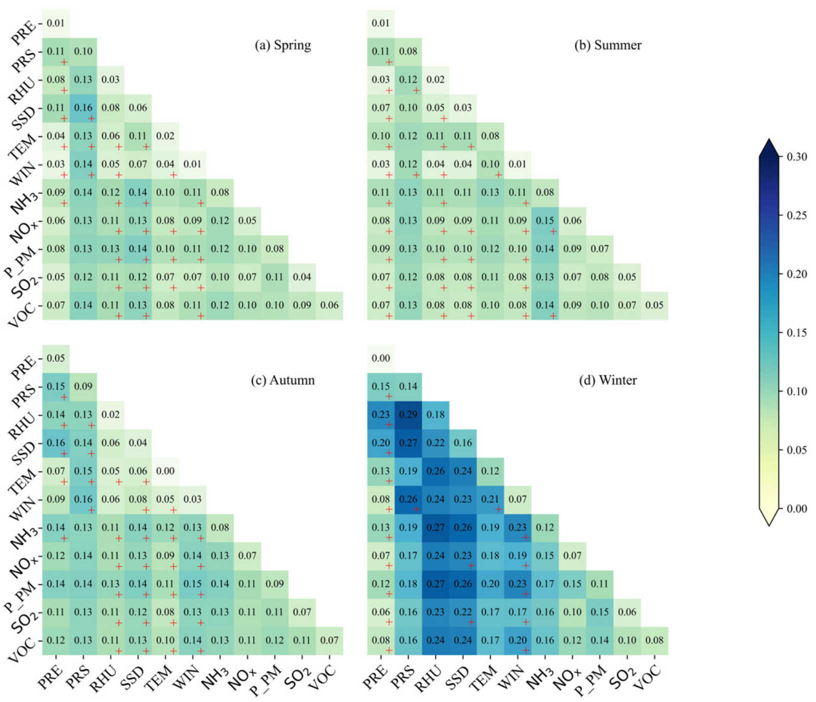


**Figure S12.** The seasonal interaction in the factors influences the spatial pattern of $PM_{2.5}$ concentrations in the Yellow River Basin. Note: "+" means that the type of interaction belongs to nonlinear enhancement, otherwise belongs to bivariate enhancement. (PRE denotes accumulated precipitation; PRS denotes surface air pressure; RHU denotes 2-m relative humidity; SSD denotes sunshine duration; TEM denotes air temperature; WIN denotes 10-m wind velocity; $NH_3$ denotes ammonia; NOx denotes nitrogen oxides; P_PM denotes primary $PM_{2.5}$; $SO_2$ denotes sulfur dioxide; VOC denotes volatile organic compounds)

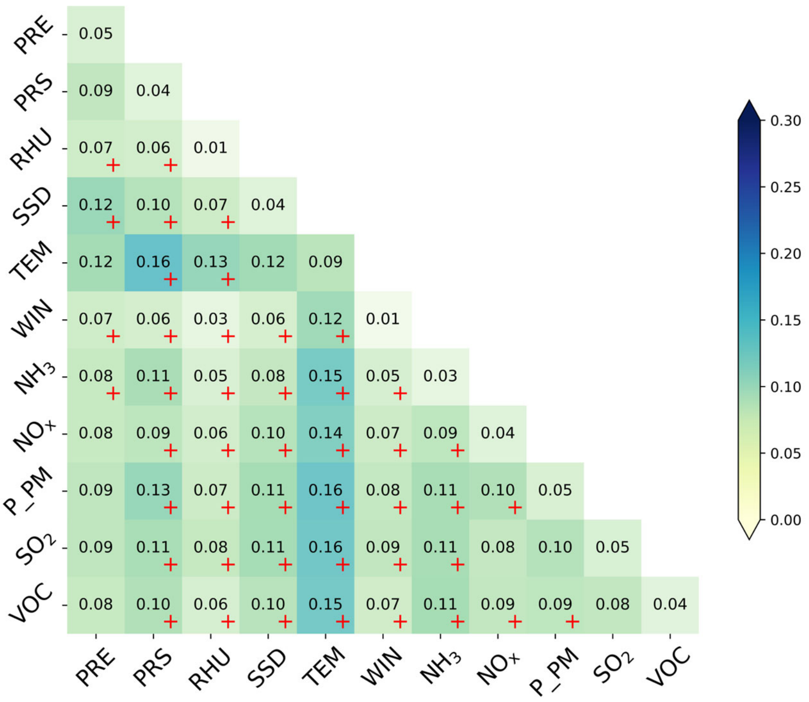

**Figure S13.** The annual interaction in the factors influences the spatial pattern of $PM_{2.5}$ concentrations in the Upper Yellow River Basin. Note: "+" means that the type of interaction belongs to nonlinear enhancement, otherwise belongs to bivariate enhancement. (PRE denotes accumulated precipitation; PRS denotes surface air pressure; RHU denotes 2-m relative humidity; SSD denotes sunshine duration; TEM denotes air temperature; WIN denotes 10-m wind velocity; $NH_3$ denotes ammonia; NOx denotes nitrogen oxides; P_PM denotes primary $PM_{2.5}$; $SO_2$ denotes sulfur dioxide; VOC denotes volatile organic compounds)

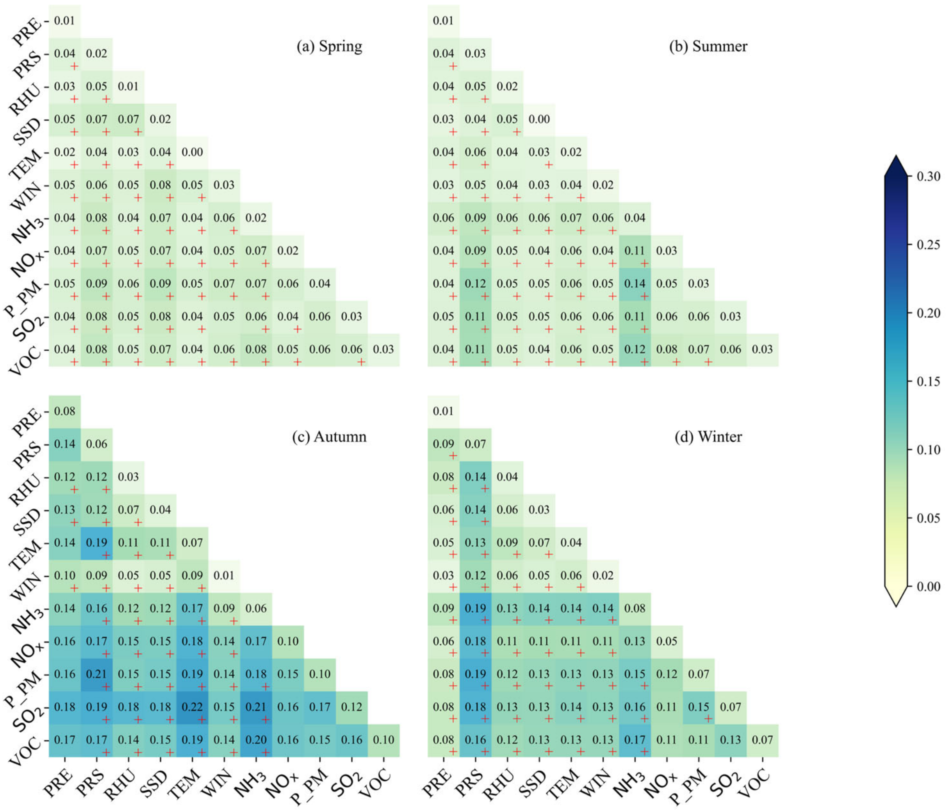


**Figure S14.** The seasonal interaction in the factors influences the spatial pattern of $PM_{2.5}$ concentrations in the Upper Yellow River Basin. Note: "+" means that the type of interaction belongs to nonlinear enhancement, otherwise belongs to bivariate enhancement. (PRE denotes accumulated precipitation; PRS denotes surface air pressure; RHU denotes 2-m relative humidity; SSD denotes sunshine duration; TEM denotes air temperature; WIN denotes 10-m wind velocity; $NH_3$ denotes ammonia; NOx denotes nitrogen oxides; P_PM denotes primary $PM_{2.5}$; $SO_2$ denotes sulfur dioxide; VOC denotes volatile organic compounds)

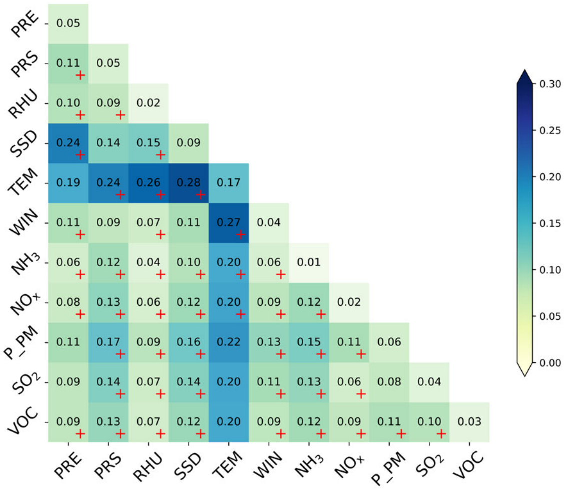


**Figure S15.** The annual interaction in the factors influences the spatial pattern of $PM_{2.5}$ concentrations in the Middle Yellow River Basin. Note: "+" means that the type of interaction belongs to nonlinear enhancement, otherwise belongs to bivariate enhancement. (PRE denotes accumulated precipitation; PRS denotes surface air pressure; RHU denotes 2-m relative humidity; SSD denotes sunshine duration; TEM denotes air temperature; WIN denotes 10-m wind velocity; $NH_3$ denotes ammonia; NOx denotes nitrogen oxides; P_PM denotes primary $PM_{2.5}$; $SO_2$ denotes sulfur dioxide; VOC denotes volatile organic compounds)

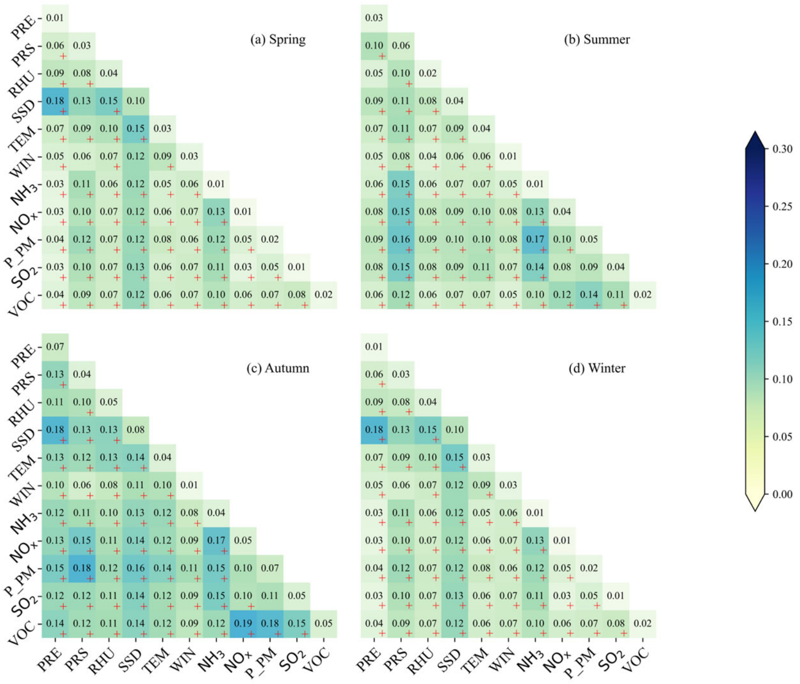


**Figure S16.** The seasonal interaction in the factors influences the spatial pattern of $PM_{2.5}$ concentrations in the Middle Yellow River Basin. Note: "+" means that the type of interaction belongs to nonlinear enhancement, otherwise belongs to bivariate enhancement. (PRE denotes accumulated precipitation; PRS denotes surface air pressure; RHU denotes 2-m relative humidity; SSD denotes sunshine duration; TEM denotes air temperature; WIN denotes 10-m wind velocity; $NH_3$ denotes ammonia; NOx denotes nitrogen oxides; P_PM denotes primary $PM_{2.5}$; $SO_2$ denotes sulfur dioxide; VOC denotes volatile organic compounds)

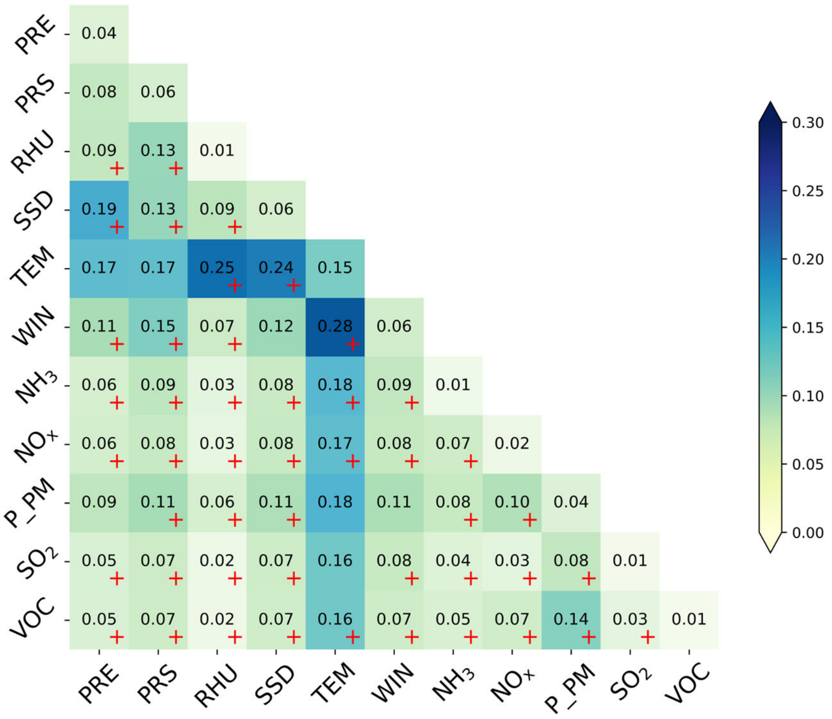


**Figure S17.** The annual interaction in the factors influences the spatial pattern of $PM_{2.5}$ concentrations in the Downstream of the Yellow River Basin. Note: "+" means that the type of interaction belongs to nonlinear enhancement, otherwise belongs to bivariate enhancement. (PRE denotes accumulated precipitation; PRS denotes surface air pressure; RHU denotes 2-m relative humidity; SSD denotes sunshine duration; TEM denotes air temperature; WIN denotes 10-m wind velocity; $NH_3$ denotes ammonia; NOx denotes nitrogen oxides; P_PM denotes primary $PM_{2.5}$; $SO_2$ denotes sulfur dioxide; VOC denotes volatile organic compounds)

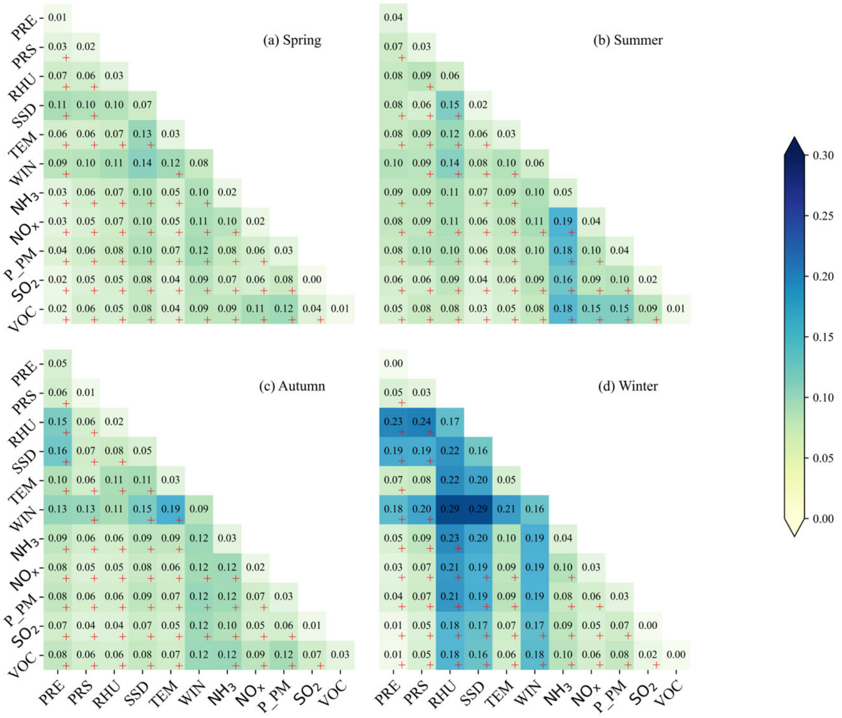

**Figure S18.** The seasonal interaction in the factors influences the spatial pattern of $PM_{2.5}$ concentrations in the Downstream of the Yellow River Basin. Note: "+" means that the type of interaction belongs to nonlinear enhancement, otherwise belongs to bivariate enhancement. (PRE denotes accumulated precipitation; PRS denotes surface air pressure; RHU denotes 2-m relative humidity; SSD denotes sunshine duration; TEM denotes air temperature; WIN denotes 10-m wind velocity; $NH_3$ denotes ammonia; NOx denotes nitrogen oxides; P_PM denotes primary $PM_{2.5}$; $SO_2$ denotes sulfur dioxide; VOC denotes volatile organic compounds)

**List of Tables**

**Table S1.** Effect of various factors on $PM_{2.5}$ concentration in the Yellow River Basin, value with *** is significant at the 0.01 level; value with ** is significant at the 0.05 level; value with * is significant at the 0.1 level. (PRE denotes accumulated precipitation; PRS denotes surface air pressure; RHU denotes 2-m relative humidity; SSD denotes sunshine duration; TEM denotes air temperature; WIN denotes 10-m wind velocity; $NH_3$ denotes ammonia; NOx denotes nitrogen oxides; P_PM denotes primary $PM_{2.5}$; $SO_2$ denotes sulfur dioxide; VOC denotes volatile organic compounds)

| Region | PRE | RHU | SSD | WIN | PRS | TEM | VOC | $NO_X$ | $NH_3$ | $SO_2$ | P_PM |
|---|---|---|---|---|---|---|---|---|---|---|---|
| Year | 0.04 *** | 0.01 *** | 0.06 *** | 0.03 *** | 0.10 *** | 0.09 *** | 0.05 *** | 0.05 *** | 0.04 *** | 0.05 *** | 0.08 *** |
| Spring | 0.01 *** | 0.03 *** | 0.05 *** | 0.01 *** | 0.10 *** | 0.02 *** | 0.06 *** | 0.06 *** | 0.07 *** | 0.04 *** | 0.07 *** |
| Summer | 0.01 *** | 0.02 *** | 0.03 *** | 0.01 *** | 0.08 *** | 0.08 *** | 0.04 *** | 0.06 *** | 0.08 *** | 0.04 *** | 0.06 *** |
| Autumn | 0.05 *** | 0.02 *** | 0.04 *** | 0.03 *** | 0.09 *** | 0.00 *** | 0.07 *** | 0.07 *** | 0.08 *** | 0.06 *** | 0.09 *** |
| Winter | 0.00 ** | 0.18 *** | 0.16 *** | 0.07 *** | 0.14 *** | 0.12 *** | 0.07 *** | 0.07 *** | 0.11 *** | 0.05 *** | 0.11 *** |

**Table S2.** Effect of various factors on $PM_{2.5}$ concentration through the whole year at the sub-basins in the Yellow River Basin, value with *** is significant at the 0.01 level; value with ** is significant at the 0.05 level; value with * is significant at the 0.1 level. (UYR denotes the Upper Yellow River Basin; MYR, denotes the Middle Yellow River Basin; DYR, denotes the Downstream of the Yellow River Basin; PRE denotes accumulated precipitation; PRS denotes surface air pressure; RHU denotes 2-m relative humidity; SSD denotes sunshine duration; TEM denotes air temperature; WIN denotes 10-m wind velocity; $NH_3$ denotes ammonia; NOx denotes nitrogen oxides; P_PM denotes primary $PM_{2.5}$; $SO_2$ denotes sulfur dioxide; VOC denotes volatile organic compounds)

| Region | PRE | RHU | SSD | WIN | PRS | TEM | VOC | $NO_X$ | $NH_3$ | $SO_2$ | P_PM |
|---|---|---|---|---|---|---|---|---|---|---|---|
| UYR | 0.05 *** | 0.01 *** | 0.04 *** | 0.01 *** | 0.04 *** | 0.09 *** | 0.03 *** | 0.03 *** | 0.02 *** | 0.04 *** | 0.05 *** |
| MYR | 0.05 *** | 0.02 *** | 0.09 *** | 0.03 *** | 0.04 *** | 0.15 *** | 0.03 *** | 0.02 *** | 0.01 *** | 0.03 *** | 0.05 *** |
| DYR | 0.04 *** | 0.01 *** | 0.06 *** | 0.06 *** | 0.05 *** | 0.14 *** | 0.00 *** | 0.02 *** | 0.01 *** | 0.01 *** | 0.04 *** |

**Table S3.** Effect of various factors on $PM_{2.5}$ concentration in spring at the sub-basins in the Yellow River Basin in spring, value with *** is significant at the 0.01 level; value with ** is significant at the 0.05 level; value with * is significant at the 0.1 level. (UYR denotes the Upper Yellow River Basin; MYR, denotes the Middle Yellow River Basin; DYR, denotes the Downstream of the Yellow River Basin; PRE denotes accumulated precipitation; PRS denotes surface air pressure; RHU denotes 2-m relative humidity; SSD denotes sunshine duration; TEM denotes air temperature; WIN denotes 10-m wind velocity; $NH_3$ denotes ammonia; NOx denotes nitrogen oxides; P_PM denotes primary $PM_{2.5}$; $SO_2$ denotes sulfur dioxide; VOC denotes volatile organic compounds)

| **Region** | **Season** | **PRE** | **RHU** | **SSD** | **WIN** | **PRS** | **TEM** | **VOC** | **$NO_X$** | **$NH_3$** | **$SO_2$** | **P_PM** |
|---|---|---|---|---|---|---|---|---|---|---|---|---|
| UYR | spring | 0.01 *** | 0.01 *** | 0.02 *** | 0.02 *** | 0.02 *** | 0.00 *** | 0.01 *** | 0.02 *** | 0.02 *** | 0.01 *** | 0.03 *** |
| MYR | spring | 0.01 *** | 0.04 *** | 0.09 *** | 0.03 *** | 0.03 *** | 0.02 *** | 0.01 *** | 0.00 *** | 0.02 *** | 0.01 *** | 0.02 *** |
| DYR | spring | 0.01 *** | 0.03 *** | 0.07 *** | 0.08 *** | 0.01 *** | 0.02 *** | 0.00 *** | 0.02 *** | 0.01 *** | 0.00 *** | 0.03 *** |

**Table S4.** Effect of various factors on $PM_{2.5}$ concentration in summer at the sub-basins in YRB in summer, value with *** is significant at the 0.01 level; value with ** is significant at the 0.05 level; value with * is significant at the 0.1 level. (UYR denotes the Upper Yellow River Basin; MYR, denotes the Middle Yellow River Basin; DYR, denotes the Downstream of the Yellow River Basin; PRE denotes accumulated precipitation; PRS denotes surface air pressure; RHU denotes 2-m relative humidity; SSD denotes sunshine duration; TEM denotes air temperature; WIN denotes 10-m wind velocity; $NH_3$ denotes ammonia; NOx denotes nitrogen oxides; P_PM denotes primary $PM_{2.5}$; $SO_2$ denotes sulfur dioxide; VOC denotes volatile organic compounds)

| **Region** | **Season** | **PRE** | **RHU** | **SSD** | **WIN** | **PRS** | **TEM** | **VOC** | **$NO_X$** | **$NH_3$** | **$SO_2$** | **P_PM** |
|---|---|---|---|---|---|---|---|---|---|---|---|---|
| UYR | summer | 0.01 *** | 0.02 *** | 0.00 *** | 0.02 *** | 0.01 *** | 0.02 *** | 0.01 *** | 0.03 *** | 0.04 *** | 0.02 *** | 0.02 *** |
| MYR | summer | 0.03 *** | 0.02 *** | 0.04 *** | 0.01 *** | 0.06 *** | 0.03 *** | 0.01 *** | 0.02 *** | 0.01 *** | 0.03 *** | 0.03 *** |
| DYR | summer | 0.04 *** | 0.06 *** | 0.02 *** | 0.06 *** | 0.03 *** | 0.03 *** | 0.00 *** | 0.04 *** | 0.04 *** | 0.01 *** | 0.03 *** |

**Table S5.** Effect of various factors on $PM_{2.5}$ concentration in autumn at the sub-basins in YRB in autumn, value with *** is significant at the 0.01 level; value with ** is significant at the 0.05 level; value with * is significant at the 0.1 level. (UYR denotes the Upper Yellow River Basin; MYR, denotes the Middle Yellow River Basin; DYR, denotes the Downstream of the Yellow River Basin; PRE denotes accumulated precipitation; PRS denotes surface air pressure; RHU denotes 2-m relative humidity; SSD denotes sunshine duration; TEM denotes air temperature; WIN denotes 10-m wind velocity; $NH_3$ denotes ammonia; NOx denotes nitrogen oxides; P_PM denotes primary $PM_{2.5}$; $SO_2$ denotes sulfur dioxide; VOC denotes volatile organic compounds)

| Region | Season | PRE | RHU | SSD | WIN | PRS | TEM | VOC | $NO_X$ | $NH_3$ | $SO_2$ | P_PM |
|---|---|---|---|---|---|---|---|---|---|---|---|---|
| UYR | autumn | 0.08 *** | 0.03 *** | 0.04 *** | 0.00 *** | 0.05 *** | 0.07 *** | 0.08 *** | 0.07 *** | 0.04 *** | 0.10 *** | 0.10 *** |
| MYR | autumn | 0.07 *** | 0.05 *** | 0.08 *** | 0.01 *** | 0.04 *** | 0.04 *** | 0.05 *** | 0.05 *** | 0.04 *** | 0.05 *** | 0.07 *** |
| DYR | autumn | 0.05 *** | 0.02 *** | 0.05 *** | 0.09 *** | 0.01 *** | 0.04 *** | 0.01 *** | 0.02 *** | 0.03 *** | 0.01 *** | 0.03 *** |

**Table S6.** Effect of various factors on $PM_{2.5}$ concentration in winter at the sub-basins in YRB in winter, value with *** is significant at the 0.01 level; value with ** is significant at the 0.05 level; value with * is significant at the 0.1 level. (UYR denotes the Upper Yellow River Basin; MYR, denotes the Middle Yellow River Basin; DYR, denotes the Downstream of the Yellow River Basin; PRE denotes accumulated precipitation; PRS denotes surface air pressure; RHU denotes 2-m relative humidity; SSD denotes sunshine duration; TEM denotes air temperature; WIN denotes 10-m wind velocity; $NH_3$ denotes ammonia; NOx denotes nitrogen oxides; P_PM denotes primary $PM_{2.5}$; $SO_2$ denotes sulfur dioxide; VOC denotes volatile organic compounds)

| Region | Season | PRE | RHU | SSD | WIN | PRS | TEM | VOC | $NO_X$ | $NH_3$ | $SO_2$ | P_PM |
|---|---|---|---|---|---|---|---|---|---|---|---|---|
| UYR | winter | 0.01 *** | 0.04 *** | 0.03 *** | 0.01 *** | 0.07 *** | 0.04 *** | 0.07 *** | 0.04 *** | 0.08 *** | 0.05 *** | 0.06 *** |
| MYR | winter | 0.00 * | 0.17 *** | 0.19 *** | 0.14 *** | 0.07 *** | 0.09 *** | 0.03 *** | 0.03 *** | 0.02 *** | 0.02 *** | 0.03 *** |
| DYR | winter | 0.00 *** | 0.17 *** | 0.15 *** | 0.16 *** | 0.03 *** | 0.05 *** | 0.00 *** | 0.02 *** | 0.04 *** | 0.00 *** | 0.03 *** |
